# Tuning the Anisotropic Thermal Transport in {110}-Silicon Membranes with Surface Resonances

**DOI:** 10.3390/nano12010123

**Published:** 2021-12-30

**Authors:** Keqiang Li, Yajuan Cheng, Maofeng Dou, Wang Zeng, Sebastian Volz, Shiyun Xiong

**Affiliations:** 1 Guangzhou Key Laboratory of Low-Dimensional Materials and Energy Storage Devices, School of Materials and Energy, Guangdong University of Technology, Guangzhou 510006, China; 20194214011@stu.suda.edu.cn; 2 Institute of Functional Nano and Soft Materials (FUNSOM), Jiangsu Key Laboratory for Carbon-Based Functional Materials and Devices, Soochow University, 199 Ren’ai Road, Suzhou 215123, China; 3 School of Physics and Materials Science, Guangzhou University, Guangzhou 510006, China; 4 Institute of Advanced Semiconductors, Hangzhou Innovation Center, Zhejiang University, Hangzhou 311200, China; maofeng.dou@zju.edu.cn; 5 Hebei Shenke Magnetic Material Co., Ltd., 9 FuDong Industrial Zone, ShiFu Avenue, Xinji 052300, China; zengwang416@163.com; 6 Laboratory for Integrated Micro Mechatronic Systems (LIMMS/CNRS-IIS), The University of Tokyo, Tokyo 153-8505, Japan; volz@iis.u-tokyo.ac.jp

**Keywords:** phonon resonance, anisotropic transport, molecular dynamics, thermal conductivity

## Abstract

Understanding the thermal transport in nanostructures has important applications in fields such as thermoelectric energy conversion, novel computing and heat dissipation. Using non-homogeneous equilibrium molecular dynamic simulations, we studied the thermal transport in pristine and resonant Si membranes bounded with {110} facets. The break of symmetry by surfaces led to the anisotropic thermal transport with the thermal conductivity along the [110]-direction to be 1.78 times larger than that along the [100]-direction in the pristine structure. In the pristine membranes, the mean free path of phonons along both the [100]- and [110]-directions could reach up to ∼100 µm. Such modes with ultra-long MFP could be effectively hindered by surface resonant pillars. As a result, the thermal conductivity was significantly reduced in resonant structures, with 87.0% and 80.8% reductions along the [110]- and [100]-directions, respectively. The thermal transport anisotropy was also reduced, with the ratio $\kappa_{110}/\kappa_{100}$ decreasing to 1.23. For both the pristine and resonant membranes, the thermal transport was mainly conducted by the in-plane modes. The current work could provide further insights in understanding the thermal transport in thin membranes and resonant structures.

## 1. Introduction

Thermal transport engineering plays an important role in many fields, ranging from thermoelectric energy conversion, novel computing with heat and heat dissipation, to heat insulation [1,2,3,4]. In the bulk state, heat transfer can be well described by Fourier’s law, with thermal conductivity (TC) being proportional to the ratio between heat flow and temperature gradient. However, when the size shrinks to the nanoscales, a new phenomenon appears and even divergent TC with system size may emerge. Due to boundary scatterings and change in phonon dispersions, the TC of materials at nanoscales differs significantly from the corresponding bulk values. When the boundary scattering dominates the transport, the TC normally decreases with the shrink in size [5,6,7]. In contrast, the TC usually increases significantly with the reduction in size when phonon dispersion variation is predominant, which is commonly observed in layered materials [8,9]. Such different size-dependent TC also provides opportunities for different applications. For example, the remarkable reduction in TC caused by boundary scattering in low dimensional materials is beneficial to thermoelectric energy conversions, where ultra-low TC is required to improve the thermoelectric figure of merit [2,10].

Recently, the phonon resonant mechanism has been widely adopted for low-TC design in low-dimensional materials [11]. Compared to the traditional scattering mechanism, the phonon resonances can block the low-frequency phonon transport efficiently with the design of small resonant structures. Another advantage of the resonant mechanism is that the resonators are usually situated on the surface of the main structure. Consequently, the geometry scattering of electrons could be minimized (the electron–electron scattering at room temperature is negligible; hence, the creation of surface pillars mainly changes electron–surface scattering processes) [11,12]. The introduction of resonant structures extensively modifies the phonon dispersion by creating numerous flat resonant modes with zero group velocity. Those resonant modes not only can reduce the group velocity by hybridizing with the original propagating modes, but can also scatter other modes by providing new scattering channels [13]. As a result, the resonant modes can reduce both the group velocity and relaxation time of propagating modes simultaneously. Phonon resonances could be produced in versatile structures, from typical pillared structures to nanojunctions, clathrates, atomic bonds and amorphous coatings [11,12,13,14,15,16,17,18,19,20,21,22,23,24,25,26,27,28,29].

In a previous work, we performed thermal transport simulations for both pristine and resonant Si membranes with different surface orientations. Among the three studied membrane types ({100}-, {110}- and {111}-membranes), the pristine {110}-membrane possessed the largest anisotropic thermal transport. To obtain more insights on the thermal transport of the {110}-membrane, we performed non-homogeneous equilibrium simulations (HNEMD) to examine the anisotropic transport in more detail. Surface resonant pillars were also introduced to reduce the anisotropic transport phenomenon.

## 2. Structures and Methods

**Figure 1:**
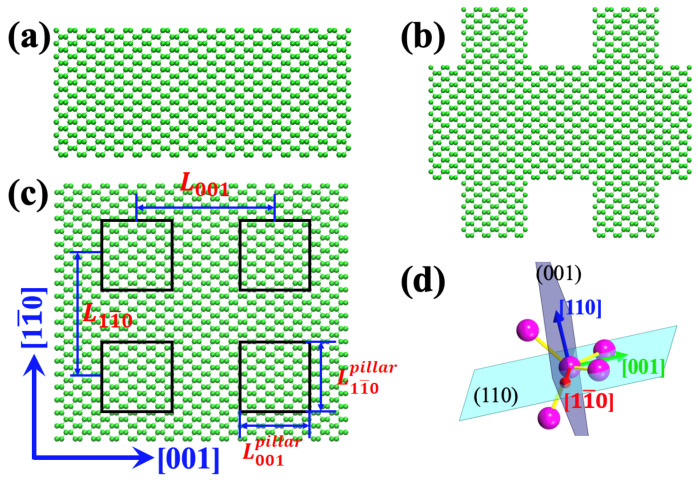
Schematic illustration of (**a**) side view of a pristine Si membrane with {110} surfaces, (**b**) side view of a resonant {110}-Si membrane, (**c**) top view of a resonant Si membrane with pillars located at the black rectangles and (**d**) relative orientations between the four bonds of a silicon atom, the {110} facet and transport directions.

Si membranes bounded with {110} facets were firstly constructed; see Figure 1a. The thickness was set as 3.8 nm. We considered the thermal transport along two orthogonal directions with low indexes, i.e., [001] and [1$\bar{1}$0]. Considering that, in cubic systems, [001] and [1$\bar{1}$0] are equivalent to [100] and [110], respectively, we hereafter use [100] and [110] as the notations for simplicity. Since the atomic configurations along the two in-plane directions [100] and [110] are not the same, which can be identified from Figure 1c, the TC along the two directions could be different and should be evaluated separately. The resonant structures were built by introducing pillars on the upper and bottom surfaces (Figure 1b). The height of pillars was set as 2 nm. The periodic length of resonant structures along the two in-plane directions was as follows: $L_{100}=4.3$ nm for the [100]-direction and $L_{110}=3.8$ nm for the [110]-direction (see Figure 1c). The cross-section size of each pillar equaled to half of the periodic length of the corresponding direction, i.e., $L_{100}^{pillar}=L_{100}/2=2.15$ nm and $L_{110}^{pillar}=L_{110}/2=1.9$ nm (Figure 1c). Consequently, the pillar density along the two directions was the same even though their size was different.

To evaluate the TC along the two in-plane directions, HNEMD [30,31], as implemented in GPUMD [32], was employed. The commonly used Stillinger–Weber potential [33] was adopted to describe the interatomic interactions among Si atoms. For all simulations, a small time step of 0.5 fs was used to eliminate the energy drift during simulations. All samples were first relaxed in the NPT ensemble for 2 ns and then shifted to the NVT ensemble for 1 ns. In the HNEMD simulations, the running TC is evaluated by recording the non-equilibrium heat flux $J_{q}$ in the NVT ensemble for 4 ns, as follows:

(1) $$\kappa \left(t\right)=\frac{1}{t}\int_{0}^{t}d\tau \frac{\langle {J_{q}\left(\tau \right)\rangle }_{\mathrm{ne}}}{TVF_{e}},$$

where *V* and *T* refer to the system volume and temperature, respectively; $F_{e}$ is the driving force parameter and $\langle {J_{q}\left(\tau \right)\rangle }_{\mathrm{ne}}$ is the non-equilibrium heat current induced by the driving force. All TCs were calculated at 300 K and averaged over four independent simulations.

To analyze the TC contribution as a function of the mode at each frequency, we decomposed the total TC into the frequency domain. The spectral TC (STC) in HNEMD simulations can be expressed as [31,34,35,36,37]

(2) $$\kappa \left(\omega \right)=\frac{2\tilde{K}\left(\omega \right)}{TVF_{e}}.$$

where $\tilde{K}\left(\omega \right)$ denotes the Fourier transform of the virial–velocity time correlation function $K\left(t\right)$, which is written as [37]

(3) $$K\left(t\right)=\sum_{i}\sum_{\alpha }\langle W_{i}\left(0\right)\cdot v_{i}\left(t\right)\rangle ,$$

where $W_{i}$ and $v_{i}$ represent the virial tensor and the velocity of atom *i*; α=x,y, and *z* denotes the Cartesian coordinate. In Equation (3), if the summation of α is taken over *x* and *y*, the contribution of TC from the in-plane modes can be distinguished (assuming that the membrane is perpendicular to the *z* direction). Similarly, if α is only summed over the *z* direction, the out-of-plane contributions to the total TC can be evaluated [31].

The mean free path (MFP) of phonons is normally distributed over a large size span, while the TC is system length-dependent when the sample length is shorter than the maximum MFP. The length dependent TC $\kappa \left(L\right)$ can be expressed as [31]

(4) $$\kappa \left(L\right)=\frac{\kappa_{diff}}{1+\lambda /L}$$

where *L* is the system length and $\kappa_{diff}$ refers to the diffusive TC, which is obtained by HNEMD simulations. λ is the phonon MFP in an infinitely long system, which is defined as the ratio between the diffusive conductivity and the ballistic conductance $G_{0}$.

(5) $$\lambda =\frac{\kappa_{diff}}{G_{0}}$$

The ballistic conductance $G_{0}$ can be obtained by NEMD simulations with ultra-short system length or by non-equilibrium Green’s function calculations [38,39].

## 3. Results and Discussion

**Figure 2:**
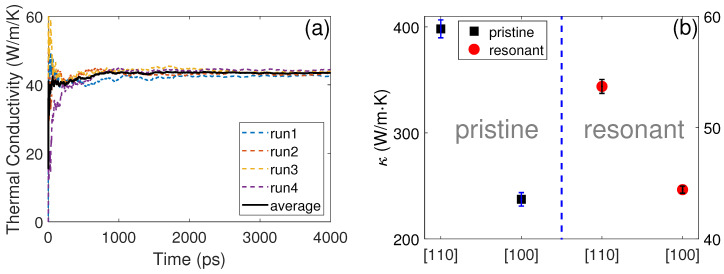
(**a**) Running TC along the [100]-direction of the resonant membrane with the variation of simulation time. (**b**) The TCs along the [100]- and [110]-directions for both the pristine and resonant membranes. The value for the pristine structures refers to the left *y*-axis and the value for the resonant structures refers to the right *y*-axis.

Figure 2a demonstrates an example (along the [100]-direction of the resonant {110}-membrane) of the running TC as a function of simulation time. Four curves corresponding to four independent simulations starting from different initial velocities are illustrated. The driving force $F_{e}$ for the pristine and resonant structures was set as 0.04 µm${}^{-1}$ and 0.4 µm${}^{-1}$. These values were tested to be small enough to keep the corresponding systems within linear response regime, i.e., leading to stable heat flux during simulations, as shown in Figure 2a. Normally, $F_{e}$ is supposed to be much smaller than the inverse of characteristic phonon MFP λ [40]. This is why the driving force of the pristine structure was much smaller than that of the resonant structures due to the MFP differences, which is demonstrated below. The driving force cannot be too small either, as a small $F_{e}$ would decrease the signal-to-noise ratio. The obtained TC along the [100] and [110] crystallographic directions for both the pristine and resonant {110}-membranes is illustrated in Figure 2b. For the pristine membranes, the TC showed large anisotropy along the [100]- (224.5 ± 28.4 W/mK) and [110] (398.2 ± 37.6 W/mK)-directions. The ratio between the TC along the [110]- and [100]-directions was $\kappa_{110}/\kappa_{100}=1.78$. The anisotropic transport of the {110}-membrane was due to the different bonding environment along the two transport directions, as shown in Figure 1c,d. With the introduction of surface pillars, the TC was dramatically reduced. The obtained TC along the [100]- and [110]-directions was 43.1 ± 1.4 and 53.2 ± 2.8 W/mK, respectively. Although the relative magnitude along the two directions followed the same trend as that of the pristine structure, the reduction percentage along the [110]-direction (87.0%) was larger than that along the [100]-direction (80.8%). Consequently, the thermal transport anisotropy was reduced to $\kappa_{110}/\kappa_{100}=1.23$. It is worth noting that the TC of resonant structures obtained with the HNEMD simulations was similar to the the value calculated with the NEMD simulations. However, the TC of the pristine membranes simulated here was larger than the NEMD results [41]. This mismatch might be due to the extrapolation issues in NEMD simulations, which is discussed below.

**Figure 3:**
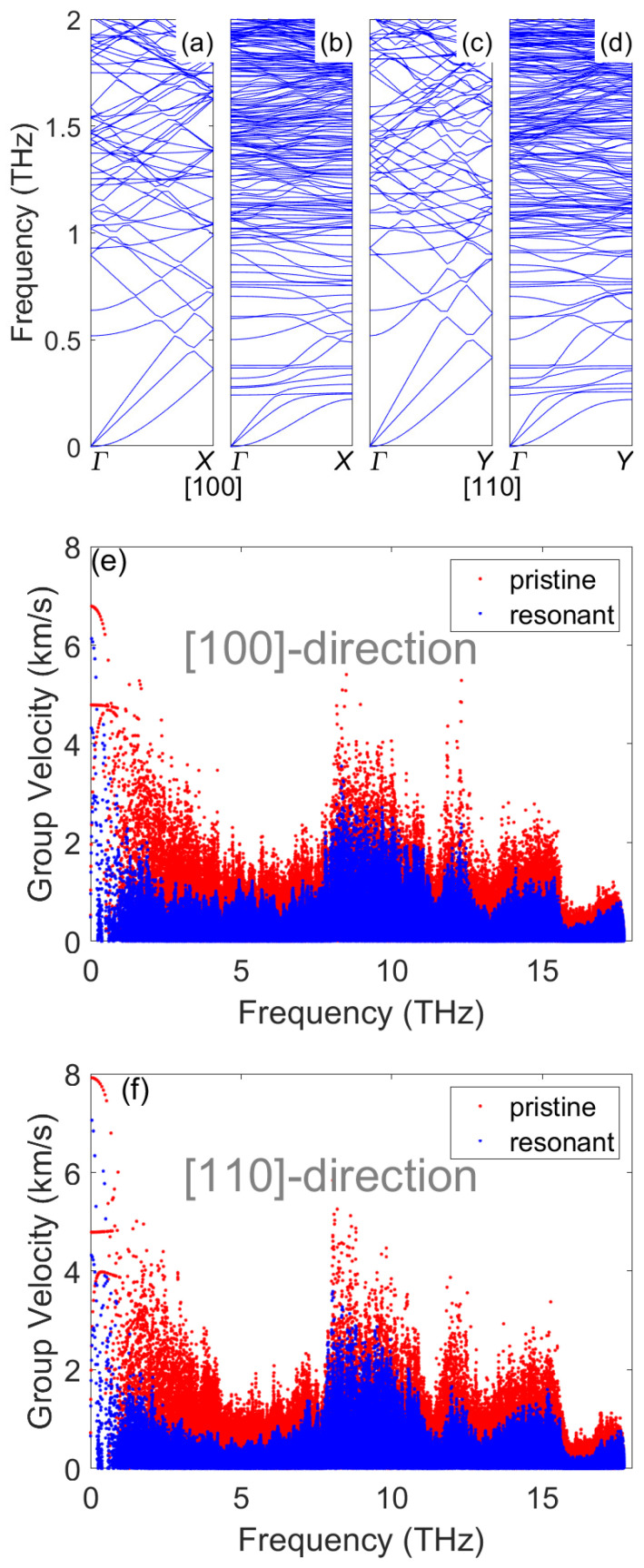
Phonon dispersion of (**a**) pristine membrane along the [100]-direction, (**b**) resonant membrane along the [100]-direction, (**c**) pristine membrane along the [110]-direction and (**d**) resonant membrane along the [110]-direction. Phonon group velocities of the pristine and resonant membranes: (**e**) along the [100]-direction and (**f**) along the [110]-direction.

To check the anisotropic transport in the pristine membrane and the significant TC reduction by resonances in more detail, we calculated the phonon dispersion curves and mode group velocities using the GULP code [42]. Figure 3a–d shows the phonon dispersion curves below 2 THz for the pristine and resonant membranes. For the pristine membrane, the out-of-plane dispersion curve (the lowest branch) along both the [100]- and [110]-directions possessed a quadratic relationship with wavevector *q*, which is a character of two-dimensional materials. Compared to the [100]-direction (Figure 3a), the three acoustic branches along the [110]-direction (Figure 3c) were steeper. As a result, the group velocity of acoustic modes along the [110]-direction was larger in the pristine membrane, which can be clearly observed from Figure 3e,f. The steeper dispersion curves may also lead to smaller phase space and consequently less scattering by other modes. The different group velocities and relaxation times of acoustic modes eventually led to large TC anisotropy.

With the creation of surface pillars, numerous flat bands were generated in the dispersion curves, as shown in Figure 3b,d. Along both the [100] and [110] crystallographic directions, the lowest resonant frequency was around 0.23 THz. Although numerous additional resonant modes were added in the dispersion curve, these modes did not contribute to heat transfer, as their group velocities were close to zero. More importantly, these flat bands could hybridize with the original propagating modes and reduce their group velocities, which can be observed from Figure 3e,f. Compared to the pristine structures, the group velocities of modes in resonant structures were reduced noticeably in the entire frequency range. Except the group velocity reduction, the existence of a flat resonant branch can dramatically increased the phonon–phonon scattering channels [13]. This is because the momentum conservation of phonon–phonon scattering can be satisfied much easier with the flat bands, as demonstrated by Wang et al. [13]. As a result, the relaxation time could also be largely reduced with the introduction of surface pillars. Finally, the reduction in both group velocity and relaxation time led to the large decrease in TC by surface pillars.

**Figure 4:**
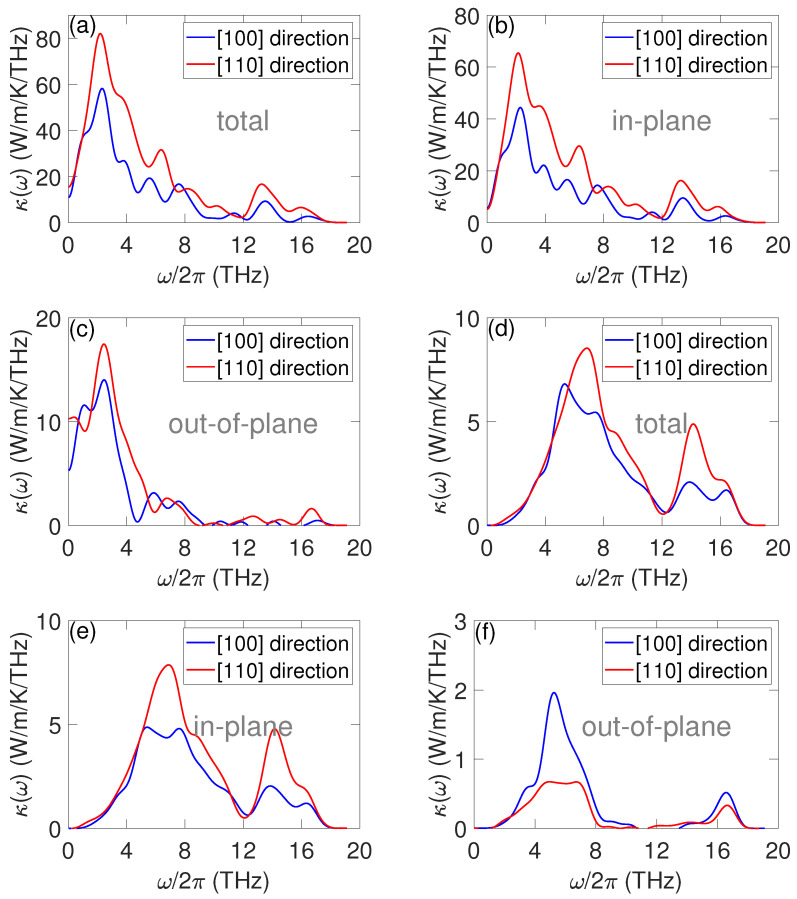
Spectrally decomposed TC for the pristine and resonant membranes. Total (**a**), in-plane (**b**) and out-of-plane (**c**) STC along the [100] and [110] crystallographic directions of the pristine membranes. Total (**d**), in-plane (**e**) and out-of-plane (**f**) STC along the [100] and [110] crystallographic directions of the resonant membranes.

To have a direct insight on the TC contribution by phonons at each frequency, we decomposed the TC in the frequency domain. The spectral TC (STC) along the [100]- and [110]-directions for both the pristine and resonant structures is shown in Figure 4. The in-plane and out-of-plane contributions, which are usually used to characterize the transport behaviors in two-dimensional materials, are also shown in Figure 4. The in-plane STC was calculated by summing only the two in-plane components (x and y in our case, since the membrane is in the xy plane) together in K(t) calculations (Equation (3)). The out-of-plane contribution was obtained by summing only the z component together in Equation (3). The out-of-plane contribution corresponds to the TC of flexural modes. In the pristine structure, the STC along the [110]-direction was larger than that along the [100]-direction in almost the entire frequency range, which was responsible for the anisotropic thermal transport in the pristine {110}-membrane. From Figure 4a, it can be observed that the TC is mostly contributed by the phonons below 10 THz along both the [100]- and [110]-directions for the pristine membrane. When separating the TC into the in-plane (Figure 4b) and out-of-plane (Figure 4c) contributions, we found that, unlike single-layered 2D materials such as graphene, where the out-of-plane TC is predominant [31], the TC of the pristine {110} Si membrane was dominated by the in-plane contributions. Such a difference was due to the reason that, in the thicker membranes studied here, the out-of-plane vibrations were suppressed, while, in graphene, the structure is very flexible and atoms can vibrate in the out-of-plane direction freely. One can image that, with the further reduction in Si membrane thickness, the relative contribution of TC from out-of-plane vibrations would increase.

Compared to the pristine membrane, a much different STC behavior was observed in the resonant membrane. From Figure 4d, one can see that the TC along both the [100]- and [110]-directions was suppressed significantly at all frequencies, which agrees with the group velocity analysis. More importantly, the reduction in TC below 3 THz by resonant pillars was much more significant than that at higher frequencies, which led to the change in STC shape, i.e., the first STC peak shifted from ∼2 THZ in the pristine structure to ∼6 THz in the resonant structure along both directions. The strong suppression of low-frequency phonon transport is also a key advantage of phonon resonances over scattering, which mainly scatters the high-frequency modes. The different frequency response regime of the resonant and scattering mechanisms make them ideal candidate to work together to tune the phonon transport in the entire frequency. Hence, they can be used to design structures with ultralow TC [13,14,27]. The strong reduction in TC at low frequencies originates from the wide spatial extension of acoustic phonons, which facilitates the overlap of resonant modes with the propagating modes, hence enlarging the scattering probability [13], while, at high frequencies, phonons are more localized and more rarely meet each other. Due to the relatively stronger TC reduction by resonant pillars at low frequencies, the high-frequency phonon contribution to the total TC is not negligible anymore. Despite the TC at high frequency was also reduced by resonant pillars, their relative magnitude at low frequencies was large. Interestingly, differing from the case in the pristine structure, the out-of-plane contribution along the [100]-direction was larger than that along the [110]-direction, which means that the resonant pillars had a larger impact on out-of-plane modes along the [110]-direction than that along the [100]-direction.

**Figure 5:**
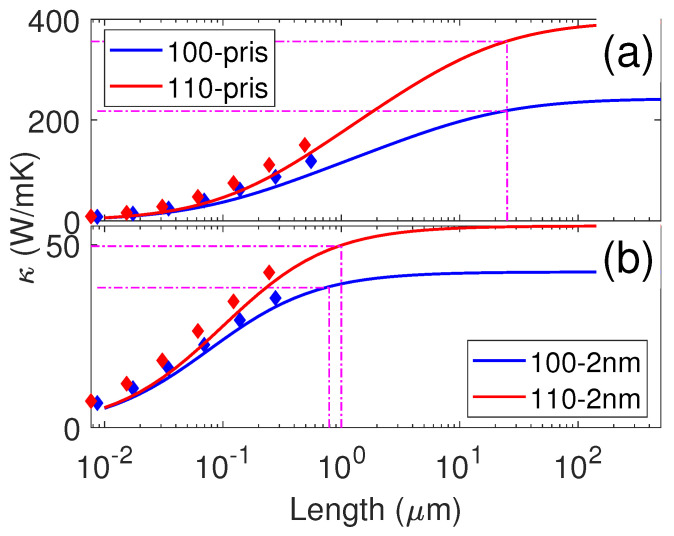
The length-dependent TC along the [100] and [110] crystallographic directions for the pristine (**a**) and resonant (**b**) membranes. The diamond symbols with the corresponding colors are the NEMD results from Ref. [41]. The horizontal dash lines denote the value of 90% of the total TC for each case and the vertical dash lines denote the corresponding length.

Based on the STC as well as ballistic thermal conductance by NEMD simulations, the length-dependent TC could also be obtained according to Equation (4). The length-dependent TC arose from the broad distribution of phonon MFPs. The calculated length-dependent TCs along the [100]- and [110]-directions for both the pristine and resonant structures are shown in Figure 5. All TCs increased with the increase in sample length due to the gradual involvement of phonons with longer MFP. For the pristine membrane, the TC saturated around 100 µm, while this length was dramatically reduced to ∼10 µm in resonant structures, indicating that the phonons with ultra-long MFP were hindered by resonant pillars. We also examined the length corresponding to 90% of the total TC (shown by the dash lines in Figure 5). The length corresponding to 90% TC in resonant membranes (∼1 µm) was also one order of magnitude smaller than that in the pristine membranes (∼22 µm), which agrees well with the driving force differences used in HNEMD simulations for the resonant and pristine membranes.

For the pristine structures, we found that the TC obtained by our HNEMD simulations was larger than that obtained by the NEMD simulations [41]. To check this differences, we also plotted the TCs from the NEMD simulations with different sample lengths. From Figure 5a, we can observe that the TC at each sample length obtained by the NEMD simulations was actually close to the HNEMD results with corresponding lengths. The variation in TC with length also followed the same trend. However, the maximum length in the NEMD simulations was ∼550 nm, which is much shorter than the maximum MFP of the pristine membrane. As a result, the linear or quadratic extrapolation would underestimate the TC of infinite long cases, as demonstrated by Dong et al. [43], who showed that using relatively short samples (compared to the maximum phonon MFP) can not make a correct extrapolation. Since HNEMD simulations revealed that the TC of the pristine membranes converges at very long lengths, one should perform NEMD simulations for samples with length of a few µm. For resonant membranes, the maximum MFP was reduced by one order of magnitude and the maximum length in the NEMD simulations was much closer to the maximum MFP. As a result, the TC obtained by NEMD and HNEMD simulations was similar along both the [100]- and [110]-directions. It is also worth mentioning that the TC obtained by NEMD simulations was slightly larger than the HNEMD results for the same length. Such small discrepancies between different simulation methods normally exist and the reason is still unclear.

## 4. Conclusions

Based on HNEMD simulations, the thermal transport in a pristine and a resonant Si membrane bounded with the {110} facet was investigated. Due to the break of symmetry by surfaces, the pristine membrane possessed anisotropic thermal transport along the two in-plane directions. The TC along the [110]-direction was 1.78 times that along the [100]-direction. Almost in the entire frequency range, the phonons along the [110]-direction contributed to a larger heat flux than that along the [100]-direction. With the creation of surface resonant pillars, the TC was significantly suppressed. The reduction percentage along the [110]-direction reached 87.0%, which was larger than that along the [100]-direction (80.8%). As a result, the thermal transport anisotropy was reduced down to the ratio $\kappa_{110}/\kappa_{100}=1.23$. The large TC reduction by resonant pillars arose from the significant hindrance of low-frequency long MFP phonons. The maximum MFP was reduced from ∼100 µm in the pristine membranes to ∼10 µm in the resonant membranes.

## Data Availability

The data presented in this study are available on request from the corresponding author.
